# Growth mindset as a structural link between parental support and academic stress among mathematically high-achieving elementary students with gifted education experience

**DOI:** 10.3389/fpsyg.2026.1954883

**Published:** 2026-10-16

**Authors:** Yile Wu, Doehee Ahn

**Affiliations:** Department of Education, Chung-Ang University, Seoul, Republic of Korea

**Keywords:** academic stress, gifted education, growth mindset, mathematically high-achieving students, parental support, secondary data analysis, self-regulated learning

## Abstract

**Introduction:**

Mathematically high-achieving elementary students with gifted education experience may experience academic stress. This study examined structural associations among perceived parental support, growth mindset, self-regulated learning, and academic stress, with supplementary exploratory comparisons of latent means across gender.

**Methods:**

Cross-sectional data from the second wave of the Korean Education Longitudinal Study 2013 (KELS 2013) were analyzed for 238 sixth-grade students who had participated in gifted education programs and answered at least 22 of 25 items correctly on a mathematics basic competency assessment. After testing measurement invariance across gender, latent mean differences were examined. Structural equation modeling controlling for gender and 5,000 bias-corrected bootstrap resamples were used to estimate direct and indirect associations.

**Results:**

Parental support was positively associated with growth mindset and self-regulated learning, and growth mindset was positively associated with self-regulated learning and negatively associated with academic stress. The direct paths from parental support and self-regulated learning to academic stress were not significant. A significant negative indirect association between parental support and academic stress through growth mindset was found. Supplementary gender comparisons indicated higher perceived parental support among boys and higher academic stress among girls.

**Discussion:**

A negative indirect association between perceived parental support and academic stress through growth mindset was observed in this sample. The measures assessed general beliefs and academic experiences. The cross-sectional findings do not establish mathematics-specific processes, temporal ordering, or causal mechanisms.

## 1 Introduction

High achievement in mathematics does not necessarily guarantee emotional well-being or smooth academic adjustment, particularly in demanding gifted-education contexts (Neihart et al., 2021; Peterson, 2009; Pfeiffer, 2012). Students with gifted education experience may have encountered advanced learning opportunities and high achievement expectations. For some students, such conditions may coexist with heightened sensitivity, perfectionistic tendencies, or social–emotional adjustment concerns documented in gifted education research (Neihart et al., 2021; Peterson, 2009). Academic stress in this subgroup should therefore be examined as a psychological adjustment issue in relation to academic demands, parental support, motivational beliefs, and self-regulatory processes (Buff et al., 2017; de la Fuente et al., 2020; Dweck, 1999, 2006; García-Ros et al., 2023; Pintrich, 2000; Zimmerman, 2002).

Academic stress concerns students’ perceived stress in relation to studying and academic demands. A transactional perspective provides a framework for understanding how such demands are appraised in relation to available coping resources (Lazarus and Folkman, 1984). In South Korean children, academic stress was negatively associated with life satisfaction and positive affect (Choi et al., 2019). Research on gifted and talented students studying at home during the COVID-19 pandemic also linked academic stress to aspects of perfectionism and feelings of mattering (Hill and Madigan, 2022). These findings support examining perceived support, motivational beliefs, and regulatory strategies in relation to academic stress among mathematically high-achieving students with gifted education experience.

Parental support has long been regarded as a contextual resource that shapes how students interpret and respond to academic demands. Emotional and academic support from parents has been linked to students’ academic functioning and lower levels of stress (Kulakow et al., 2021; Unger et al., 2000). In gifted-education contexts, supportive parental attitudes may also help create an environment in which high achievement expectations are accompanied by opportunities for appropriate challenge and growth rather than only by sources of pressure (Jung and Lee, 2024).

To clarify how parental support relates to academic stress, two student-level processes are especially relevant: growth mindset and self-regulated learning. Growth mindset shapes how students interpret academic difficulty, particularly whether setbacks are viewed as evidence of fixed ability or as opportunities for development (Dweck, 1999, 2006; Dweck and Leggett, 1988). Self-regulated learning captures the strategic processes through which students plan, monitor, and regulate cognition, motivation, and behavior during learning (Pintrich, 2000; Zimmerman, 2002, 2008). Examining these processes together may help explain why academic demands are experienced as more or less stressful across students (de la Fuente et al., 2020; García-Ros et al., 2023).

Gender differences in parental support, growth mindset, self-regulated learning, and academic stress have been reported inconsistently across studies. Some studies have found no gender differences in perceived parental support, whereas others have reported higher levels among girls (Colarossi and Eccles, 2000; Kristjánsson and Sigfúsdóttir, 2009; Ringeisen and Raufelder, 2015). Findings for growth mindset and self-regulated learning have also varied by academic domain, developmental stage, and sample characteristics (Bezzina, 2010; Dan et al., 2024; Malespina et al., 2022; Tsai, 2023; Yao et al., 2024). Studies of university students have reported higher perceived stress among female than male students (Bermejo-Franco et al., 2022; Graves et al., 2021). Yet these gender patterns have rarely been tested simultaneously in high-achieving elementary students with gifted education experience.

The present study addresses a specific question about academic stress: how are perceived parental support, beliefs about the developability of intelligence, and regulatory strategies jointly related to stress within a selected high-achieving elementary-school sample? Prior work has linked parental support to stress and growth mindset to self-regulated learning, but the implications of these associations for academic stress in this subgroup require clarification (Bai et al., 2021; Kulakow et al., 2021). Modeling these constructs together allows their conditional associations with stress to be distinguished and permits examination of indirect associations involving motivational beliefs, regulatory strategies, or both.

The contribution is a theory-guided assessment of whether this pattern of associations is consistent with an account connecting perceived environmental resources, students’ beliefs, and academic stress. The model does not establish a new causal mechanism or test whether parental support moderates the association between mindset and stress. Supplementary gender comparisons describe variation in perceived resources and stress within this subgroup; they do not test gender differences in structural paths.

## 2 Theoretical background and research questions

### 2.1 Parental support as a contextual resource

Parental support has been widely recognized as a contextual resource that shapes how students interpret and respond to academic demands. Parents provide learning-related resources and also shape the social and emotional context of children’s learning experience (Buff et al., 2017). Prior studies have linked parental autonomy support and authoritative parenting to stronger growth mindset beliefs (Chen et al., 2025; Damrongpanit, 2022; Kim et al., 2017). Parental support has also been associated with higher self-regulated learning, including students’ resistance to distraction, effort regulation, and strategic learning behaviors (Choe, 2020; Lee et al., 2012). In gifted education, the home psychological environment may be especially relevant to self-regulated learning (Park et al., 2011). Together, this evidence positions parental support as a contextual resource related to students’ motivational beliefs and self-regulated learning.

Parental support may also be associated with academic stress. Studies involving adolescents and middle school students have reported that higher parental support is linked to lower academic stress (Kulakow et al., 2021; Toraman et al., 2022). Less is known, however, about whether these associations hold when parental support, growth mindset, self-regulated learning, and academic stress are modeled simultaneously among mathematically high-achieving elementary students with gifted education experience. The present study therefore tested both direct and indirect links between parental support and academic stress.

### 2.2 Growth mindset and self-regulated learning as motivational-regulatory factors

Growth mindset refers to the belief that intelligence and ability can be developed through effort and effective learning strategies and is considered an important motivational belief in academic settings (Dweck, 1999, 2006; Dweck and Leggett, 1988). Students with a stronger growth mindset are more likely to interpret failure and challenge as opportunities for learning rather than as evidence of limited ability. Prior studies have found that growth mindset is positively related to self-regulated learning strategy use (Bai and Guo, 2021; Bai et al., 2021), and research with gifted students has reported similar links between beliefs about the developability of intelligence and self-regulated learning strategies (Shin and Ahn, 2014). Within a social-cognitive account, beliefs about the developability of ability may orient students toward sustained effort and the revision of learning strategies, providing a theoretical rationale for placing growth mindset before self-regulated learning in the proposed model (Dweck and Leggett, 1988; Zimmerman, 2002). Growth mindset has also been associated with more adaptive responses to academic challenges and lower perceived stress (Mosanya, 2021; Zhao et al., 2021), including during periods of educational transition (Zhao et al., 2023).

The specificity of the mindset measure is important. Han and Borgonovi (2026) distinguished beliefs about general intelligence from domain-specific mindsets and found that associations with achievement varied across academic and creative domains. Accordingly, a general intelligence mindset measure should not be treated as a measure of mathematics-specific beliefs. In the present study, mathematics achievement defined the analytic subgroup, whereas the mindset scale assessed general beliefs about the developability of intelligence.

Cultural context may also qualify the interpretation of mindset findings. Using PISA 2018 data from 48 countries, Wang et al. (2026) reported lower endorsement of growth mindsets and weaker associations with reading and science achievement in less WEIRD (Western, educated, industrialized, rich, and democratic) contexts. These findings concern academic achievement and do not directly establish cultural differences in the mindset–stress association. Whether general growth mindset is associated with academic stress among mathematically high-achieving Korean elementary students with gifted education experience remains an empirical question.

Self-regulated learning describes how learners set goals, monitor progress, and regulate cognition, motivation, and behavior during learning (Pintrich, 2000; Zimmerman, 2002, 2008). Cognitive regulation concerns the processing and monitoring of learning, including rehearsal, elaboration, organization, and metacognition. Behavioral regulation concerns the management of learning activity and resources, including effort, time, study environment, and help-seeking (Pintrich, 2000; Zimmerman, 2002). These functions may have different implications for stress: cognitive strategies may support understanding and monitoring, whereas behavioral strategies may help organize responses to academic demands. These are theoretical possibilities rather than separately tested pathways in the present study. The structural model examined the variance shared by cognitive and behavioral regulation through a higher-order self-regulated learning factor.

Prior studies have linked stronger self-regulated learning with more effective planning and monitoring and with lower academic stress (de la Fuente et al., 2020; Marsch et al., 2024). These relations may not generalize uniformly across developmental stage, academic domain, or achievement level. Students in gifted education programs may encounter academic demands and achievement expectations that differ from those experienced by general student populations (Neihart et al., 2021; Pfeiffer, 2012). This subgroup therefore offers a useful context for testing how self-regulated learning relates to growth mindset and academic stress.

### 2.3 Academic stress as perceived strain in the academic context

A transactional perspective conceptualizes stress in relation to how individuals appraise demands and their resources for coping with them (Lazarus and Folkman, 1984). Applied to academic settings, this account suggests that similar learning demands may be associated with different levels of perceived stress. Parental support can be viewed as an available contextual resource, growth mindset as a belief potentially relevant to the interpretation of difficulty, and self-regulated learning as a set of strategies for managing learning demands. These roles provide a theoretical basis for examining their direct and indirect associations with academic stress.

The KELS academic stress scale assesses students’ reported stress concerning their studies. It does not directly measure primary appraisal, secondary appraisal, or the adequacy of coping resources. The transactional perspective therefore serves as an interpretive framework rather than a process model fully tested by the available measures. The proposed associations also allow for the possibility that strategy use alone may be insufficient when students perceive demands as exceeding available support.

### 2.4 Gender differences in parental support, growth mindset, self-regulated learning, and academic stress

Gender differences in parental support, growth mindset, self-regulated learning, and academic stress have been examined, but findings remain mixed. Some studies have found no gender differences in perceived parental support, whereas others have reported higher levels among girls (Colarossi and Eccles, 2000; Kristjánsson and Sigfúsdóttir, 2009; Ringeisen and Raufelder, 2015). Evidence on growth mindset is similarly inconclusive, with findings varying across gifted and general education contexts (Malespina et al., 2022; Tsai, 2023; Yao et al., 2024). For self-regulated learning, several studies have reported higher strategy use among girls, including in mathematics or elementary-school samples (Bezzina, 2010; Dan et al., 2024). Among university students, female students have reported higher perceived stress than male students (Graves et al., 2021). Whether a similar pattern occurs in academic stress among the present elementary-school subgroup requires empirical examination.

Exploratory latent mean comparisons were used to describe whether boys and girls in this subgroup differed in perceived parental support, growth mindset, cognitive regulation, behavioral regulation, and academic stress. Differences in perceived support and stress are relevant to understanding variation in students’ academic experiences within a selected high-achieving group. No directional hypotheses were specified for these comparisons because prior findings vary across populations and domains. Gender was included as a covariate in the pooled structural model to adjust for gender-related variation. These analyses address mean-level differences and adjusted associations; they do not test moderation of structural paths by gender.

### 2.5 Present study

The primary aim was to examine structural associations among perceived parental support, growth mindset, self-regulated learning, and academic stress among mathematically high-achieving elementary students with gifted education experience. Parental support was specified as an upstream contextual resource because supportive family interactions may provide a setting for the development of students’ beliefs and learning practices (Chen et al., 2025; Choe, 2020). Growth mindset was positioned before self-regulated learning because beliefs about the developability of ability may guide engagement with learning strategies (Bai et al., 2021; Dweck and Leggett, 1988). Academic stress was the focal adjustment outcome.

This ordering represents a theoretically motivated specification. Alternative accounts are also plausible: learning experiences may shape mindset, and academic stress may alter strategy use or perceptions of parental support. Cross-sectional data cannot determine which ordering best represents change over time. Mathematics achievement was used to define the sample, whereas the psychological measures concerned general beliefs and academic experiences. The model therefore addresses general academic associations within this selected subgroup. Supplementary exploratory latent mean comparisons followed measurement invariance testing across gender, and the pooled structural model included gender as a covariate.

The research questions and hypothesized model are presented in Figure 1.

**Figure 1:**
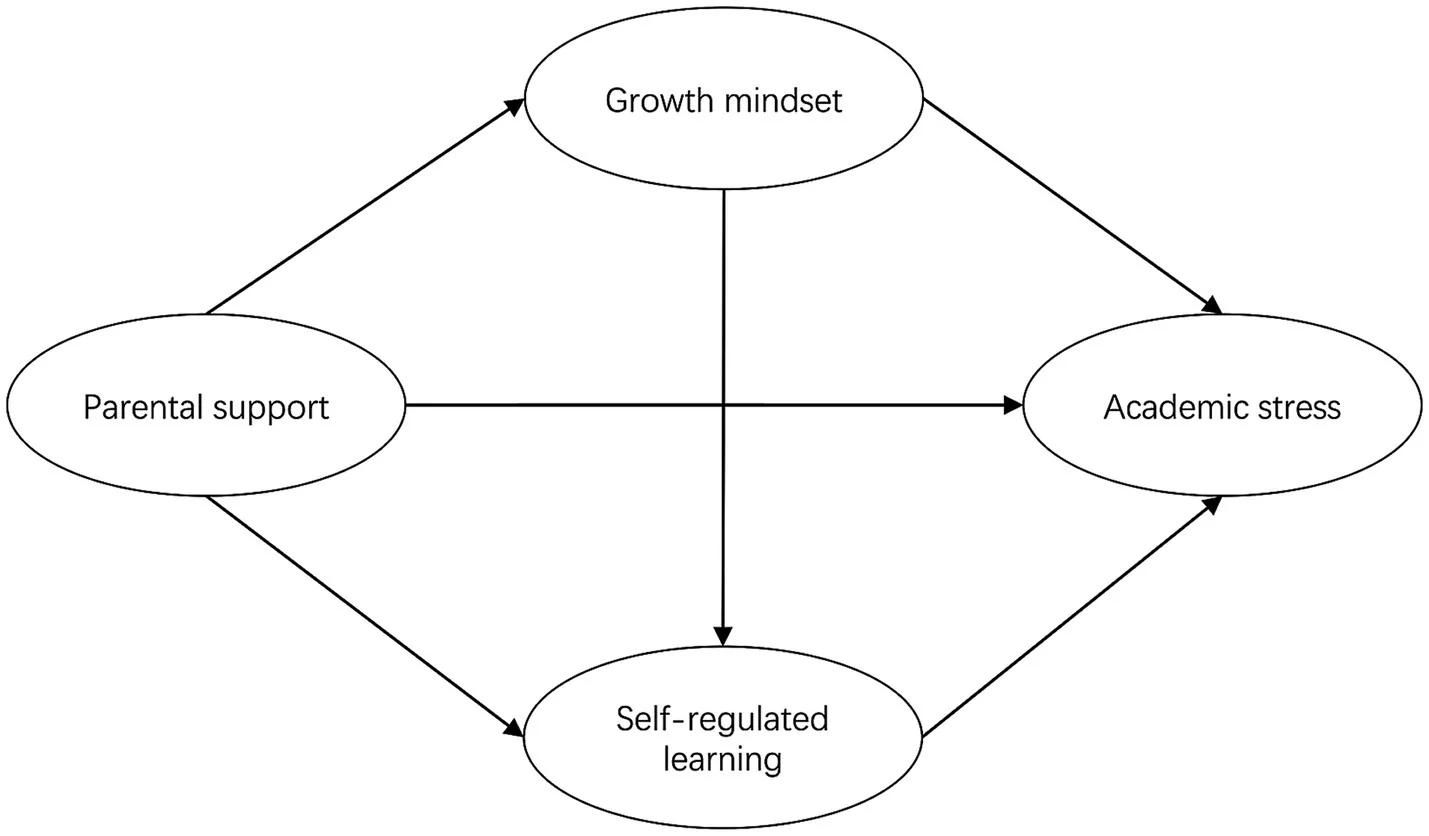
Research model. The figure depicts the hypothesized structural model. In the structural analyses, gender was included as a covariate but is not displayed in the figure for visual clarity.

*RQ1*: How are perceived parental support, growth mindset, self-regulated learning, and academic stress structurally related after controlling for gender?

*RQ2*: Are the indirect associations between parental support and academic stress through growth mindset, self-regulated learning, or both statistically significant?

*RQ3*: As a supplementary exploratory analysis, are there gender differences in the latent means of parental support, growth mindset, cognitive regulation, behavioral regulation, and academic stress?

## 3 Materials and methods

### 3.1 Data source and participants

This study was based on a secondary analysis of data from the Korean Education Longitudinal Study 2013 (KELS 2013), conducted by the Korean Educational Development Institute (KEDI). The second wave of KELS 2013 was administered in 2014, and data from sixth-grade elementary school students were used in the present study. The analytic sample was identified through a sequential screening procedure (see Figure 2). Of the initial KELS 2013 sample of 7,324 students, 5,783 had valid questionnaire data. Among these students, 355 reported having participated in a gifted education program. The mathematics achievement criterion was then applied: students who answered at least 22 of the 25 items correctly on the mathematics basic competency test were retained. Of the 355 students with gifted education experience, 238 met this criterion, whereas 117 did not. All 238 students who met the mathematics achievement criterion had complete data on parental support, growth mindset, self-regulated learning, academic stress, and gender. Accordingly, the final analytic sample comprised 238 students.

**Figure 2:**
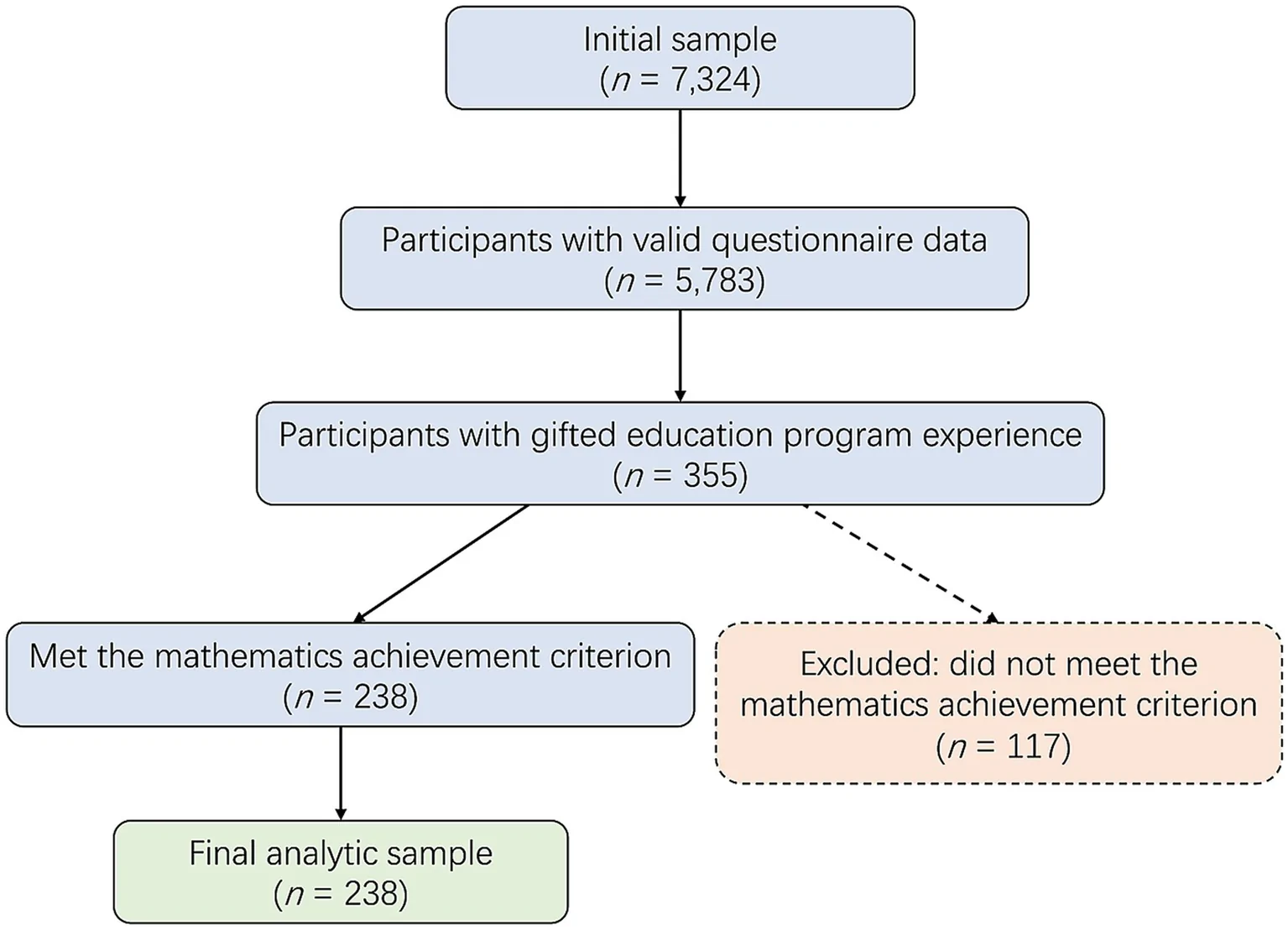
Flowchart of the sample selection process.

The sample size was determined by the number of eligible cases available in this secondary dataset (*N* = 238). The final measurement model included 19 observed indicators and a higher-order self-regulated learning construct, and the supplementary gender comparisons involved 137 boys and 101 girls. Sample-size requirements for SEM depend on model complexity, factor loadings, and the magnitude of the associations being estimated (Wolf et al., 2013). To make estimation precision transparent, structural path estimates are reported with standard errors, and indirect associations are reported with 95% bias-corrected confidence intervals based on 5,000 bootstrap resamples (Tables 1, 2). Acceptable model fit and consistency across sensitivity analyses do not establish adequate statistical power. Given the available sample and the modest, unequal gender-group sizes, small indirect associations and latent mean differences require cautious interpretation, and non-significant findings should not be taken as evidence that the corresponding associations or differences are absent.

**Table 1:** Unstandardized and standardized structural path estimates for the hypothesized model.

Path	*B*	*β*	SE	C. R.
Parental support→Growth mindset	0.149	0.246^**^	0.049	3.063
Parental support→Self-regulated learning	0.261	0.476^***^	0.046	5.717
Parental support→Academic stress	−0.149	−0.121	0.118	−1.263
Growth mindset→Self-regulated learning	0.351	0.388^***^	0.062	5.622
Growth mindset→Academic stress	−0.600	−0.296^***^	0.174	−3.452
Self-regulated learning→Academic stress	−0.416	−0.185	0.235	−1.767

*N* = 238. The structural equation model included gender as a covariate. Covariate path estimates are not shown. ^**^p < 0.01, ^***^p < 0.001, SE, standard error; C. R., critical ratio.

**Table 2:** Specific indirect associations between parental support and academic stress through growth mindset and self-regulated learning.

Predictor variables		Mediator variables		Outcome variables	*B*	SE	95% CI
Parental support	→	Growth mindset	→	Academic stress	−0.090^**^	0.049	[−0.234, −0.021]
Parental support	→	SRL	→	Academic stress	−0.108	0.090	[−0.312, 0.041]
Parental support	→	Growth mindset→SRL	→	Academic stress	−0.022	0.027	[−0.102, 0.006]

*N* = 238, ^**^p < 0.01, SRL, Self-regulated learning. Indirect associations were estimated using 5,000 bootstrap resamples and 95% bias-corrected confidence intervals; an indirect association was considered statistically significant when the confidence interval did not include zero. The path through both growth mindset and SRL represents a specific indirect association within the hypothesized model and does not imply temporal ordering.

Because the analytic sample was defined by both gifted education program participation and mathematics achievement, the SEM results should be interpreted as model-based estimates for this specific subgroup rather than as population-representative estimates for all Korean sixth-grade students. Sampling weights and school-level clustering were not incorporated into the AMOS models; this limitation is addressed in the Discussion.

The mathematics criterion of 22 correct responses represented 88% of the maximum test score. Students meeting this criterion and reporting gifted education experience were operationally described as mathematically high-achieving elementary students with gifted education experience. This definition reflects achievement on the KELS mathematics basic competency test and reported program participation; it does not establish formal identification as mathematically gifted.

Among the 238 students, 137 (57.6%) were boys and 101 (42.4%) were girls. Gender was coded from the demographic information provided in KELS 2013 and was available only as a binary category (boys and girls). As the dataset did not include additional gender categories, findings related to gender should be interpreted within this limitation. The regional distribution of the sample was 41 students (17.2%) from metropolitan areas, 79 (33.2%) from large cities, 84 (35.3%) from small and medium-sized cities, and 34 (14.3%) from rural areas. In terms of school type, 213 students (89.5%) attended public schools and 25 (10.5%) attended private schools. Mathematics basic competency test scores were distributed as follows: 52 students (21.8%) answered 22 items correctly, 53 (22.3%) answered 23 items correctly, 84 (35.3%) answered 24 items correctly, and 49 (20.6%) answered all 25 items correctly (see Table 3).

**Table 3:** Participants’ demographic characteristics.

Characteristic		*N*	%
Gender	Male	137	57.6
	Female	101	42.4
Region	Special metropolitan city	41	17.2
	Large city	79	33.2
	Small- and medium-sized city	84	35.3
	Rural area	34	14.3
School type	National/public	213	89.5
	Private	25	10.5
Mathematics test score (number correct)	22	52	21.8
	23	53	22.3
	24	84	35.3
	25	49	20.6
Total		238	100.0

*N* = 238.

### 3.2 Measures

The major study variables were derived from the second-wave KELS 2013 student survey and mathematics basic competency test data. Parental support, growth mindset, self-regulated learning, and academic stress were measured using student self-report items. For all measures, higher scores reflected higher levels of the corresponding construct. Details of the alternative scale specifications, item-removal criteria, reliability estimates, measurement-model results, and sensitivity analyses are reported in Supplementary Tables S1–S9 and described in the sections below.

#### 3.2.1 Mathematics achievement criterion

Mathematics basic competency test scores from the second wave of KELS 2013 were used to identify the mathematically high-achieving group. The test consisted of 25 items. Among students who participated in gifted education programs, those who answered 22 or more items correctly were classified as mathematically high-achieving. This cutoff corresponded to 88% of the total test score. To examine the robustness of the findings to the selection criterion, an additional sensitivity analysis was conducted using a more stringent cutoff of 23 correct responses.

#### 3.2.2 Perceived parental support

Perceived parental support was assessed using the parental support scale included in KELS 2013. The original scale consisted of eight items. A sample item is, “My parents give me advice about how to study.” Responses were recorded on a 5-point Likert scale ranging from 1 (strongly disagree) to 5 (strongly agree), with higher scores indicating higher levels of perceived parental support.

Three items with standardized loadings below 0.50 were excluded. In addition, one item was excluded because its content referred to the broader home educational environment rather than specific parental support behaviors. To reduce the risk of relying on a single data-driven measurement specification, the original eight-item, five-item, and final four-item specifications were compared. Model fit, validity, and reliability indices for these alternative specifications are reported in Supplementary Table S1, and the corresponding structural-model sensitivity analyses are reported in Supplementary Table S9. The final measure consisted of four items. The four-item scale showed acceptable internal consistency (Cronbach’s α = 0.757).

#### 3.2.3 Growth mindset

Growth mindset was assessed using the KELS 2013 growth mindset scale, which was developed from the mindset framework of Dweck and Leggett (1988). The scale comprised three items. A sample item is, “Anyone can become smarter through effort.” Responses were recorded on a 4-point Likert scale ranging from 1 (strongly disagree) to 4 (strongly agree). Higher scores indicated higher levels of growth mindset.

Because the scale included only three items, the one-factor confirmatory factor analysis (CFA) model was saturated (*df* = 0), and overall model fit indices were not evaluated. Instead, standardized factor loadings, average variance extracted (AVE), composite reliability (CR), and Cronbach’s α were examined. Cronbach’s α for the three-item scale was 0.855. Supplementary Table S2 presents AVE, CR, and Cronbach’s α for the three-item scale.

#### 3.2.4 Self-regulated learning

Self-regulated learning was assessed using the cognitive regulation and behavioral regulation strategy scales included in KELS 2013. These scales assess cognitive and self-regulatory processes conceptually related to those examined using the Motivated Strategies for Learning Questionnaire (MSLQ; Pintrich and De Groot, 1990). The cognitive-regulation scale initially comprised 12 items covering rehearsal, elaboration, organization, and metacognition. Behavioral regulation comprised academic performance regulation, effort regulation, time management, space management, and help-seeking/resource use. One help-seeking/resource-use item was removed at the subscale CFA stage because its standardized factor loading was below 0.50, leaving 19 behavioral-regulation items. The rehearsal subscale was subsequently excluded from the final cognitive-regulation factor, as explained below.

Sample items include “When studying, I make sure that I remember what I have learned” for cognitive regulation and “I can identify and carry out the tasks that I need to complete” for behavioral regulation. Responses were recorded on a 4-point Likert scale ranging from 1 (strongly disagree) to 4 (strongly agree), with higher scores indicating greater use of self-regulated learning strategies. Cronbach’s α coefficients for the subscales were 0.800 for rehearsal, 0.817 for elaboration, 0.778 for organization, 0.866 for metacognition, 0.886 for academic performance regulation, 0.841 for effort regulation, 0.814 for time management, 0.674 for space management, and 0.846 for help-seeking/resource use. Cronbach’s α was 0.882 for the initial 12-item cognitive-regulation scale, which included rehearsal, and 0.929 for the 19-item behavioral-regulation scale. After excluding rehearsal, Cronbach’s α for the retained cognitive-regulation items was 0.903. CFA results for the initial 12-item cognitive-regulation scale and the behavioral-regulation scale before and after deletion of the low-loading item are presented in Supplementary Table S3. AVE, CR, and Cronbach’s α values for each subscale are reported in Supplementary Tables S4–S6.

In the overall SEM, self-regulated learning was modeled as a higher-order latent construct represented by cognitive regulation and behavioral regulation. Cognitive regulation was indicated by elaboration, organization, and metacognition, whereas behavioral regulation was indicated by academic performance regulation, effort regulation, time management, space management, and help-seeking/resource use. Separate structural paths from cognitive regulation and behavioral regulation to academic stress were not estimated. The disturbance variance of behavioral regulation, representing variance not explained by higher-order self-regulated learning, was fixed at 0.00001. Estimates involving the higher-order self-regulated learning factor should therefore be interpreted conditional on this near-zero variance constraint.

The rehearsal subscale was excluded from the final cognitive-regulation component because its standardized loading in the overall measurement model was below 0.50. Its internal consistency was acceptable (Cronbach’s *α* = 0.800), indicating that weak representation of the broader cognitive-regulation factor should be distinguished from inconsistency among the rehearsal items. Rehearsal emphasizes repetition and retention, whereas elaboration, organization, and metacognition concern other aspects of processing and monitoring. This distinction offers a possible interpretation of the lower loading, but the present data cannot determine whether it reflects developmental characteristics, sample-specific measurement properties, or limitations of the higher-order model. Exclusion narrows the content represented by the final cognitive-regulation factor and does not imply that rehearsal is educationally unimportant. Models including and excluding rehearsal were compared in the sensitivity analyses (Supplementary Tables S8, S9).

#### 3.2.5 Academic stress

Academic stress was assessed using the academic stress scale included in KELS 2013. The scale consisted of four items. A sample item is, “I feel stressed when my studies do not go well.” Responses were recorded on a 5-point Likert scale ranging from 1 (strongly disagree) to 5 (strongly agree). Higher scores indicated higher levels of academic stress. Cronbach’s α for the four-item scale was 0.819. CFA results, AVE, CR, and Cronbach’s α for the academic stress measure are presented in Supplementary Table S7.

### 3.3 Analytical procedures

The analyses proceeded in several steps. First, descriptive statistics, including means, standard deviations, skewness, and kurtosis, were computed for all study variables. Skewness and kurtosis were evaluated using the criteria suggested by West et al. (1995). Because all major variables were measured using student self-report questionnaires, Harman’s single-factor test (Harman, 1976) was conducted as an initial diagnostic check of potential common method bias. As this procedure alone cannot rule out common method bias, the findings were interpreted with consideration of the limitations associated with self-report data.

Second, latent correlation analyses were conducted to examine the associations among perceived parental support, growth mindset, self-regulated learning, and academic stress. Discriminant validity was evaluated using the criterion proposed by Fornell and Larcker (1981), by comparing the square root of the average variance extracted (AVE) for each latent construct with the correlations among latent constructs. Convergent validity was assessed based on standardized factor loadings, critical ratios (C. R.), average variance extracted (AVE), and composite reliability (CR). Standardized loadings ≥ 0.50, |C. R.| > 1.965, AVE ≥ 0.50, and CR ≥ 0.70 indicated acceptable convergent validity (Hair et al., 2010).

Third, structural equation modeling (SEM) was used to examine the structural associations among perceived parental support, growth mindset, self-regulated learning, and academic stress while controlling for gender. Parameters were estimated using maximum likelihood estimation. Model fit was evaluated using χ^2^/*df*, the comparative fit index (CFI), the Tucker-Lewis index (TLI), the root mean square error of approximation (RMSEA), and the standardized root mean square residual (SRMR). The adopted screening thresholds were CFI and TLI ≥ 0.90 and RMSEA and SRMR ≤ 0.08. Model fit was interpreted jointly across indices and cautiously in light of the more stringent combinations of cutoffs discussed by Hu and Bentler (1999). All analyses were conducted using AMOS 30.0.

Fourth, the significance of the indirect associations linking perceived parental support and academic stress through growth mindset and self-regulated learning was examined. Indirect associations were evaluated using 95% bias-corrected bootstrap confidence intervals based on 5,000 bootstrap resamples. An indirect association was considered statistically significant when the confidence interval did not include zero. Phantom variables were used to evaluate the significance of specific indirect associations. Because the study was based on cross-sectional secondary data, indirect associations were interpreted as structural indirect associations rather than temporal or causal mediation effects.

Fifth, sensitivity analyses were conducted to examine the robustness of the primary findings. First, to evaluate the influence of alternative specifications of perceived parental support, the final four-item model was compared with a five-item model (excluding only the three items with standardized factor loadings below 0.50) and the original eight-item model. Second, to assess the influence of alternative specifications of self-regulated learning, models excluding and including the rehearsal subscale were compared. Third, to examine the impact of the mathematics achievement criterion, the primary analyses based on students who answered 22 or more mathematics test items correctly were compared with supplementary analyses using a more stringent criterion of 23 or more correct responses. Across sensitivity analyses, changes in the direction, magnitude, and statistical significance of the major structural paths and indirect associations were examined. Detailed model specifications, fit indices, key path coefficients, and indirect association results are reported in Supplementary Table S9.

Finally, configural, metric, and scalar measurement invariance across gender was evaluated sequentially using χ^2^-difference tests and changes in CFI, RMSEA, and SRMR. Changes were calculated as the fit index for the more constrained model minus that for the preceding, less constrained model. A decrease in CFI of no more than 0.010 was considered a practical benchmark for retaining invariance (Cheung and Rensvold, 2002). Additional benchmarks were increases in RMSEA of no more than 0.015 and in SRMR of no more than 0.030 for metric invariance and 0.010 for scalar invariance (Chen, 2007). These benchmarks were interpreted jointly with the χ^2^-difference tests and the modest, unequal group sizes.

Under the retained scalar invariance model, latent means of perceived parental support, growth mindset, cognitive regulation, behavioral regulation, and academic stress were compared across gender. Cognitive and behavioral regulation were examined separately at the first-order level. In the full-sample SEM, these two factors represented a higher-order self-regulated learning construct through which structural associations were examined. The gender comparisons were supplementary and did not test gender differences in structural paths.

## 4 Results

### 4.1 Single-factor diagnostic for shared method variance

The largest factor in Harman’s single-factor diagnostic accounted for 33.597% of the variance. This descriptive result was not treated as evidence that common method bias was absent or negligible. Shared self-report measurement may have influenced the observed associations, and this diagnostic cannot determine the extent of such bias (Podsakoff et al., 2003).

### 4.2 Descriptive statistics

Table 4 presents the means, standard deviations, skewness, and kurtosis of the study variables. Skewness ranged from −1.738 to 0.543, and kurtosis ranged from −0.469 to 4.796. These univariate summaries describe the observed score distributions but do not establish multivariate normality.

**Table 4:** Descriptive statistics of study variables.

Variable	*M*	SD	Skewness	Kurtosis
Parental support	3.497	0.914	−0.350	−0.203
Growth mindset	3.620	0.511	−1.738	4.796
Self-regulated learning	3.131	0.463	−0.238	0.001
Academic stress	2.359	1.026	0.543	−0.469

*N* = 238. The self-regulated learning score used for these descriptive statistics includes rehearsal, whereas rehearsal was excluded from the final measurement model and the cognitive-regulation scores reported in Table 9.

### 4.3 Correlations among variables

Table 5 presents the latent correlations among the four constructs. Perceived parental support was positively associated with growth mindset (φ = 0.250, *p* < 0.01) and self-regulated learning (φ = 0.557, *p* < 0.001), and negatively associated with academic stress (φ = −0.341, *p* < 0.001). Growth mindset was positively associated with self-regulated learning (φ = 0.506, *p* < 0.001) and negatively associated with academic stress (φ = −0.428, *p* < 0.001). Self-regulated learning was also negatively associated with academic stress (φ = −0.403, *p* < 0.001).

**Table 5:** Correlations among latent variables and the square roots of AVE.

Latent variable	1	2	3	4
1. Parental support	(0.670)			
2. Growth mindset	0.250^**^	(0.823)		
3. Self-regulated learning	0.557^***^	0.506^***^	(0.945)	
4. Academic stress	−0.341^***^	−0.428^***^	−0.403^***^	(0.733)

*N* = 238, ^**^p < 0.01, ^***^p < 0.001. Diagonal values in parentheses indicate the square roots of AVE, and off-diagonal values represent correlations (φ) among latent variables. The square root of AVE for self-regulated learning was calculated from the final measurement model, which excluded rehearsal.

Discriminant validity was examined by comparing the latent correlations (φ) with the square roots of the average variance extracted (AVE) values for each construct, following the criterion proposed by Fornell and Larcker (1981). As shown in Table 5, the absolute values of the latent correlations ranged from 0.250 to 0.557, whereas the square roots of the AVE values ranged from 0.670 to 0.945. Because all latent correlations were lower than the corresponding square roots of the AVE values, evidence of discriminant validity was supported.

### 4.4 Measurement model and validity evidence

Before examining the structural associations among the study variables, the measurement model was evaluated. The initial measurement model, which included the rehearsal factor, showed acceptable fit to the data, χ^2^(163) = 287.818, *p* < 0.001, CFI = 0.943, TLI = 0.933, RMSEA = 0.057, and SRMR = 0.058. However, the rehearsal factor, a subdimension of cognitive regulation, showed a standardized factor loading below 0.50 and was therefore excluded from the final measurement model. After removing the rehearsal factor, the final measurement model demonstrated acceptable fit, χ^2^(145) = 262.872, *p* < 0.001, CFI = 0.945, TLI = 0.935, RMSEA = 0.059, and SRMR = 0.055. A comparison of model fit before and after removal of the rehearsal factor is reported in Supplementary Table S8.

Convergent validity was assessed using standardized factor loadings, critical ratios (C. R.), average variance extracted (AVE), and composite reliability (CR). Standardized factor loadings in the final measurement model were above the 0.50 criterion, and composite reliability values were within acceptable ranges (see Table 6). The AVE values for growth mindset, cognitive regulation, behavioral regulation, and academic stress met the recommended 0.50 criterion. The AVE for perceived parental support was 0.448, slightly below the recommended threshold; therefore, evidence for the convergent validity of perceived parental support should be interpreted with caution. Nevertheless, the standardized loadings and composite reliability value for perceived parental support were acceptable, HTMT results supported discriminant validity, and sensitivity analyses yielded largely consistent results. Therefore, perceived parental support was retained in the final model.

**Table 6:** Factor loadings, convergent validity, and composite reliability for the measurement model.

Construct/indicator	*B*	*β*	SE	C. R.	AVE	Composite reliability
Parental support						
Support 1	1.000	0.641	-	-	0.448	0.762
Support 2	1.151	0.695^***^	0.141	8.178		
Support 3	1.096	0.779^***^	0.127	8.619		
Support 4	0.768	0.541^***^	0.113	6.785		
Growth mindset						
Mindset 1	1.000	0.848	-	-	0.677	0.862
Mindset 2	0.992	0.875^***^	0.069	14.472		
Mindset 3	0.956	0.740^***^	0.077	12.391		
Cognitive regulation						
Elaboration	1.000	0.756	-	-	0.649	0.847
Organization	1.101	0.812^***^	0.088	12.448		
Metacognition	1.129	0.847^***^	0.087	12.977		
Behavioral regulation						
Academic performance regulation	1.000	0.821	-	-	0.550	0.858
Effort regulation	0.774	0.731^***^	0.063	12.306		
Time management	1.061	0.790^***^	0.078	13.640		
Space management	0.776	0.585^***^	0.083	9.321		
Help-seeking and resource use	0.920	0.757^***^	0.071	12.881		
Higher-order self-regulated learning						
Cognitive regulation	1.000	0.887	-	-	0.893	0.944
Behavioral regulation	1.125	1.000^***^	0.099	11.307		
Academic stress						
Stress 1	1.000	0.717	-	-	0.537	0.822
Stress 2	1.086	0.760^***^	0.108	10.101		
Stress 3	0.774	0.696^***^	0.082	9.404		
Stress 4	0.930	0.755^***^	0.093	10.047		
Recommended cutoffs		≥0.50		> 1.965	≥0.50	≥0.70

*N* = 238, ^***^p < 0.001. *B*, unstandardized estimate; *β*, standardized factor loading; SE, standard error; C. R., critical ratio; AVE, average variance extracted. Recommended cutoffs were standardized factor loading ≥ 0.50, |C. R.| > 1.965, AVE ≥ 0.50, and CR ≥ 0.70 (Hair et al., 2010).

To further examine discriminant validity, the heterotrait–monotrait ratio (HTMT) was calculated (Henseler et al., 2015). As shown in Table 7, HTMT values ranged from 0.189 to 0.471, well below the recommended cutoff value of 0.90. These results provided additional support for discriminant validity among the latent constructs.

**Table 7:** Discriminant validity results based on the heterotrait–monotrait ratio (HTMT).

Construct	1	2	3	4
1. Parental support	-			
2. Growth mindset	0.189	-		
3. Self-regulated learning	0.471	0.446	-	
4. Academic stress	0.254	0.368	0.345	-

*N* = 238.

### 4.5 Structural model and direct associations

The fit of the structural equation model controlling for gender as a covariate was examined. The proposed model showed acceptable fit to the data, χ^2^(161) = 289.345, *p* < 0.001, CFI = 0.941, TLI = 0.930, RMSEA = 0.058, and SRMR = 0.061 (see Figure 3).

**Figure 3:**
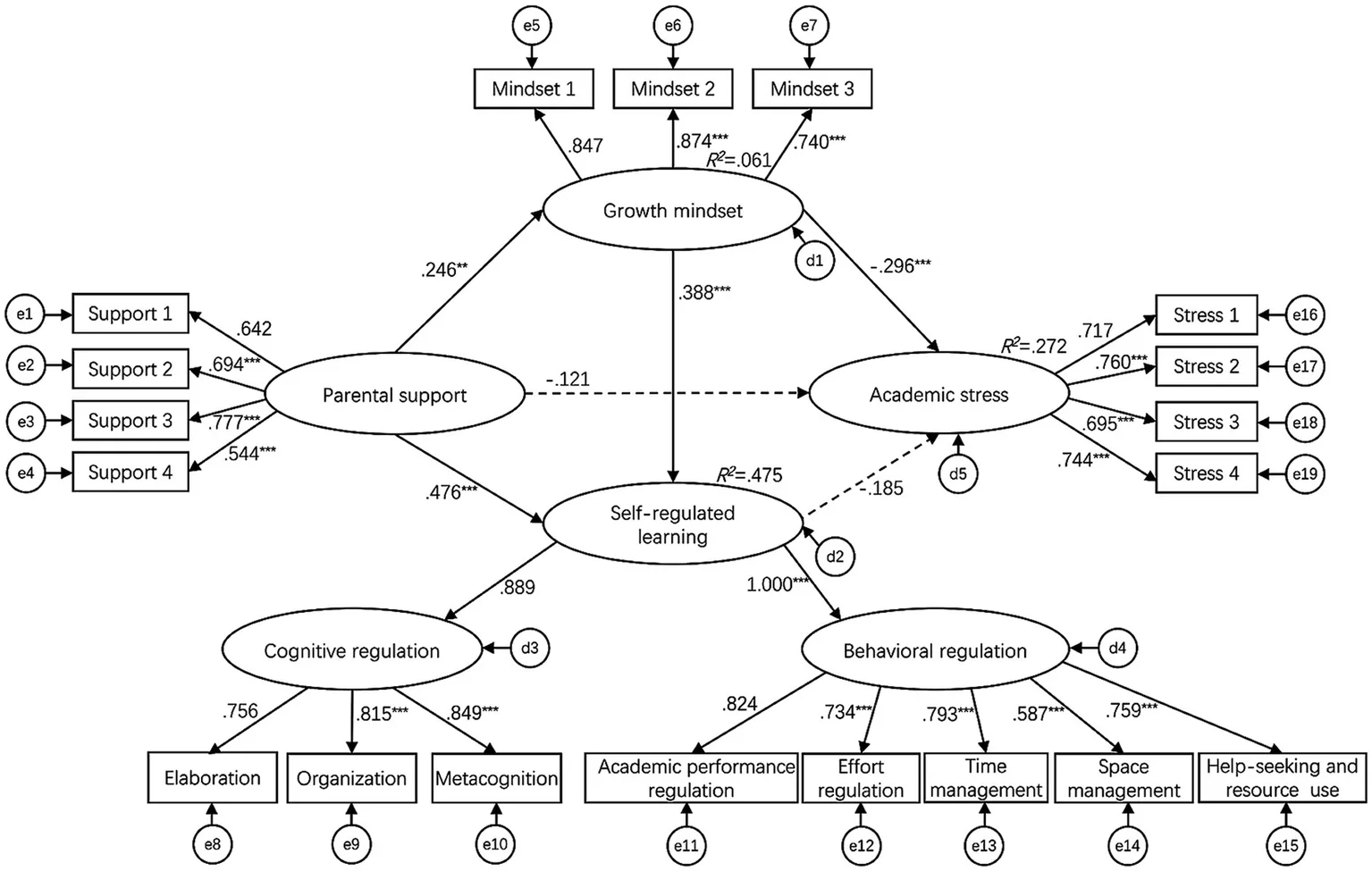
Standardized path estimates for the hypothesized structural model of academic stress. *N* = 238. Standardized path coefficients are shown for the structural equation model with gender included as a covariate. Covariate paths are omitted for visual clarity. Solid lines indicate significant paths (^**^p < 0.01, ^***^p < 0.001), and dashed lines indicate non-significant paths (*p* > 0.05). *R*^2^ values represent the proportion of variance explained in each endogenous construct.

Within the structural model controlling for gender, perceived parental support was positively associated with growth mindset (*β* = 0.246, *p* < 0.01) and self-regulated learning (*β* = 0.476, *p* < 0.001). Growth mindset was positively associated with self-regulated learning (*β* = 0.388, *p* < 0.001) and negatively associated with academic stress (*β* = −0.296, *p* < 0.001). In contrast, no significant direct association was found between perceived parental support and academic stress, nor between self-regulated learning and academic stress (see Table 1). The final model explained 6.1% of the variance in growth mindset, 47.5% of the variance in self-regulated learning, and 27.2% of the variance in academic stress.

### 4.6 Indirect pathways

Indirect pathways were tested using 5,000 bias-corrected bootstrap resamples. The path through growth mindset was significant and negative, indicating an indirect association between perceived parental support and academic stress (B = −0.090, 95% CI [−0.234, −0.021]). The SRL-only path (B = −0.108, 95% CI [−0.312, 0.041]) and the sequential path through growth mindset and SRL (B = −0.022, 95% CI [−0.102, 0.006]) were not significant (see Table 2). Thus, only the pathway through growth mindset was supported. Given the cross-sectional design, this result should be interpreted as a structural association rather than temporal mediation.

### 4.7 Sensitivity analyses

Robustness checks compared the primary model with alternative parental-support specifications, an SRL model including rehearsal, and a stricter mathematics achievement criterion. Most alternative models showed acceptable fit. However, the original eight-item parental-support model did not meet the prespecified CFI and TLI criteria, although its RMSEA and SRMR values remained within the adopted thresholds (CFI = 0.882, TLI = 0.865, RMSEA = 0.072, and SRMR = 0.079). Despite this poorer measurement specification, the main structural pattern was stable: parental support remained positively associated with growth mindset, and growth mindset remained negatively associated with academic stress. The indirect association through growth mindset was also significant across all alternative specifications, including the stricter mathematics criterion (*N* = 186, B = −0.064, 95% CI [−0.211, −0.003]). Detailed results are reported in Supplementary Table S9.

### 4.8 Supplementary exploratory gender comparisons

Latent mean analyses examined gender differences in perceived parental support, growth mindset, cognitive regulation, behavioral regulation, and academic stress. Before comparing latent means, configural, metric, and scalar invariance across gender were evaluated.

The comparison between the configural and metric invariance models was not statistically significant, Δχ^2^(14) = 19.862, *p* = 0.135. The comparison between the metric and scalar invariance models was also not statistically significant, Δχ^2^(14) = 18.043, *p* = 0.205. Changes in CFI, RMSEA, and SRMR were within the adopted practical benchmarks for both comparisons, supporting metric and scalar invariance (see Table 8). The supplementary latent mean comparisons were interpreted cautiously given the modest, unequal group sizes.

**Table 8:** Measurement invariance across gender.

Model	Invariance level	*χ* ^2^	*df*	CFI	RMSEA	SRMR	△CFI	△RMSEA	△SRMR
Model 1	Configural invariance	477.682	284	0.913	0.054	0.0598	-	-	-
Model 2	Metric invariance	497.544	298	0.910	0.053	0.0608	−0.003	−0.001	0.0010
Model 3	Scalar invariance	515.587	312	0.908	0.053	0.0613	−0.002	0.000	0.0005

*N* = 238 (137 boys and 101 girls). Changes in fit indices were calculated as the more constrained model minus the preceding, less constrained model: Model 2 minus Model 1 for metric invariance and Model 3 minus Model 2 for scalar invariance. Negative ΔCFI values indicate poorer fit, whereas positive ΔRMSEA and ΔSRMR values indicate poorer fit.

In the scalar invariance model reported in Table 8, factor loadings and observed-variable intercepts were constrained to equality across gender. The five latent means were fixed to zero in the reference group (boys) and freely estimated in the girls’ group. The model yielded χ^2^(312) = 515.587, *p* < 0.001, CFI = 0.908, TLI = 0.900, RMSEA = 0.053, and SRMR = 0.0613.

As shown in Table 9, significant gender differences were observed for perceived parental support and academic stress. Male students reported higher levels of perceived parental support than female students (*p* < 0.01), whereas female students reported higher levels of academic stress than male students (*p* < 0.001). No significant gender differences were found for growth mindset, cognitive regulation, or behavioral regulation. For descriptive interpretation, Cohen’s *d* was calculated from the observed group means using the pooled standard deviation. Following Cohen (1992), values of 0.20, 0.50, and 0.80 were interpreted as small, medium, and large effects, respectively. The effect sizes for perceived parental support (*d* = 0.407) and academic stress (*d* = −0.459) were between the conventional small and medium benchmarks.

**Table 9:** Latent mean differences by gender.

Latent variables		Boys		Girls		Cohen’s *d*	Total *M*
		Latent *M*	*M*	Latent *M*	*M*		
Parental support		0	3.652	−0.357^**^	3.287	0.407	3.497
Growth mindset		0	3.647	−0.056	3.584	0.123	3.620
SRL	Cognitive regulation	0	3.192	−0.002	3.168	0.042	3.182
	Behavioral regulation	0	3.203	−0.015	3.185	0.037	3.195
Academic stress		0	2.164	0.478^***^	2.624	−0.459	2.359

*N*_Boy_ = 137, *N*_Girl_ = 101, ^**^p < 0.01, ^***^p < 0.001, SRL, Self-regulated learning. Latent means were fixed to zero in the boys’ group; estimates for the girls’ group represent unstandardized latent mean differences calculated as girls minus boys. Cohen’s *d* was calculated as the observed mean for boys minus that for girls, divided by the pooled within-group standard deviation. Cognitive-regulation scores exclude rehearsal, consistent with the final measurement model.

## 5 Discussion

The discussion first considers the structural associations among perceived parental support, growth mindset, self-regulated learning, and academic stress after controlling for gender. It then examines the indirect associations between parental support and academic stress through growth mindset, self-regulated learning, or both, followed by the supplementary exploratory comparisons of latent means by gender. These findings are interpreted in relation to motivational beliefs and academic adjustment within the selected sample.

### 5.1 Structural associations among parental support, growth mindset, self-regulated learning, and academic stress

Parental support was linked to stronger growth mindset and self-regulated learning, whereas growth mindset was linked to lower academic stress. Direct paths from parental support and self-regulated learning to stress were not significant after the other variables were considered. Growth mindset showed a significant negative association with academic stress in the specified model. This finding is consistent with accounts linking beliefs about ability to responses to academic challenge, but it does not establish that mindset is more important than parental support or regulatory strategies (Dweck and Leggett, 1988; Zimmerman, 2002).

The positive association between parental support and growth mindset is consistent with research linking parental autonomy support and authoritative parenting to students’ growth mindset beliefs (Chen et al., 2025; Damrongpanit, 2022; Kim et al., 2017). Parental support was also positively associated with self-regulated learning, aligning with studies showing that academic and emotional support from parents is related to self-regulated learning and academic adjustment (Choe, 2020; Lee et al., 2012). Evidence from gifted-education research further suggests that the home environment may be particularly relevant to self-regulated learning among gifted learners (Park et al., 2011). For students facing advanced expectations, parental support may therefore provide both emotional reassurance and a learning context in which effort, strategy use, and persistence are emphasized.

Growth mindset was also related to greater use of self-regulated learning strategies, extending prior evidence on mindset and strategic learning to this sample. Previous studies have shown that students with stronger growth mindset beliefs tend to use self-regulated learning strategies more frequently (Bai and Guo, 2021; Bai et al., 2021), and research involving gifted students has similarly reported positive associations between beliefs about the malleability of intelligence and self-regulated learning strategy use (Shin and Ahn, 2014).

The negative association between growth mindset and academic stress is compatible with the possibility that beliefs about developable ability relate to less threatening interpretations of academic difficulty. However, appraisals and objective academic demands were not measured, so this explanation remains theoretical. Prior studies link schoolwork and career concerns to adolescents’ reported stress and academic stress to lower well-being among South Korean children (Kim, 2021; Choi et al., 2019). These findings provide context for the present associations without identifying the processes responsible for them.

The observed association concerns general intelligence beliefs and general academic stress within a subgroup selected using mathematics achievement and gifted education experience. It cannot be assumed to describe mathematics-specific mindset or stress processes (Han and Borgonovi, 2026). Cross-national research suggests that associations between growth mindset and academic achievement vary across cultural contexts (Wang et al., 2026). These findings concern achievement and do not directly establish cultural differences in the association between growth mindset and academic stress. The present results should be interpreted within the Korean educational context, with caution when generalizing to other cultural settings. Because the present study did not compare cultural groups, it cannot establish that the observed pattern is specific to Korea.

The bivariate associations of parental support and self-regulated learning with academic stress were negative, whereas their direct paths in the full model were not statistically significant. These differences concern marginal and conditional associations and do not demonstrate that the corresponding relationships are absent or weaker than the mindset–stress association. Previous studies have reported negative associations between parental support and academic stress (Kulakow et al., 2021) and between self-regulated learning and academic stress (de la Fuente et al., 2020). The non-significant direct path from self-regulated learning to academic stress may also be related to the measurement of self-regulated learning in the present study. The construct was represented primarily by cognitive and behavioral regulation strategies and did not include motivational regulation, although self-regulated learning is generally conceptualized as encompassing cognitive, motivational, and behavioral regulation (Pintrich, 2000; Zimmerman, 2002, 2008).

### 5.2 Indirect pathways through growth mindset and self-regulated learning

The indirect-path results sharpen this interpretation. Perceived parental support was linked to lower academic stress through growth mindset, whereas the SRL-only and sequential pathways were not significant. A cautious reading is that supportive family contexts may matter for stress insofar as they are aligned with beliefs that frame academic difficulty as improvable rather than fixed. This interpretation is consistent with studies linking parental autonomy support and supportive parenting practices to students’ growth mindset (Chen et al., 2025; Damrongpanit, 2022; Kim et al., 2017), as well as research describing growth mindset as a motivational belief that shapes responses to failure and academic challenge (Dweck, 1999, 2006; Dweck and Leggett, 1988). It also aligns with studies showing that growth mindset is associated with lower stress or more adaptive adjustment under academic challenge (Mosanya, 2021; Zhao et al., 2021; Zhao et al., 2023).

Robustness checks supported this pattern across variations in the parental-support scale, the SRL specification, and the mathematics achievement cutoff. The pathway through growth mindset remained significant under the stricter criterion of at least 23 correct responses on the 25-item mathematics test (*N* = 186, B = −0.064, 95% CI [−0.211, −0.003]), providing additional evidence that the main indirect association was not specific to the primary selection threshold (see Supplementary Table S9).

These indirect paths should not be read as developmental sequences. Cross-sectional data cannot establish whether parental support precedes growth mindset or whether lower stress fosters more adaptive beliefs. Methodological work on mediation has warned that cross-sectional mediation models can provide biased estimates of longitudinal mediation processes and cannot establish temporal ordering (Cole and Maxwell, 2003; Maxwell and Cole, 2007). Longitudinal and intervention studies are therefore needed to test temporal ordering and causal mechanisms.

### 5.3 Supplementary exploratory gender comparisons

Boys reported higher perceived parental support, whereas girls reported higher academic stress. No statistically significant latent mean differences were detected for growth mindset, cognitive regulation, or behavioral regulation. These results describe mean-level differences and do not indicate whether structural associations vary by gender. The parental-support result should be read against mixed prior findings, including studies reporting no gender differences in perceived parental support and others reporting higher parental support among girls (Colarossi and Eccles, 2000; Kristjánsson and Sigfúsdóttir, 2009; Ringeisen and Raufelder, 2015). Because the present study did not assess observed parental behaviors or parent–child interaction patterns, the psychosocial processes underlying this gender difference cannot be determined.

The non-significant latent mean differences in growth mindset, cognitive regulation, and behavioral regulation should be interpreted alongside the mixed findings reported in previous studies. Tsai (2023) reported no significant gender differences in growth mindset among gifted elementary students, whereas other studies have found gender differences that varied by domain and sample characteristics (Malespina et al., 2022; Yao et al., 2024). Similarly, although girls have often reported greater use of self-regulated learning strategies than boys in mathematics or elementary-school contexts (Bezzina, 2010; Dan et al., 2024), such differences were not observed in the present sample. This pattern suggests that gender differences in motivational and regulatory constructs may depend on developmental stage, domain, achievement level, and sample composition.

Girls’ higher academic stress is consistent with findings on school stress among adolescents (García-Moya et al., 2025). Studies of university students also provide evidence of gender differences in stress, although their developmental and educational contexts differ from those of the present sample (Bermejo-Franco et al., 2022; Day and Livingstone, 2003; Graves et al., 2021). It is also consistent with evidence that school-related stress contributes to gender differences in adolescent mental health (Högberg et al., 2020). In Korea’s achievement-oriented educational context, where long study hours and examination pressure have been documented, this pattern may reflect both individual stress experiences and broader expectations surrounding academic performance (Lee and Larson, 2000). This gender difference should nevertheless be interpreted cautiously. The data did not include coping strategies, academic expectations, or observed parental behaviors; therefore, girls’ higher stress should not be attributed to emotional sensitivity or vulnerability. Although gender differences in interpersonal stress, emotional reactivity, and rumination have been discussed in previous research, these mechanisms were not directly tested here (Nolen-Hoeksema, 2012; Rose and Rudolph, 2006). The binary gender variable available in KELS also limits the scope of interpretation. Future research should examine whether these differences are linked to academic expectations, perfectionism, parent–child communication, and coping strategies.

### 5.4 Limitations and future directions

The present study has several limitations that should be considered when interpreting the findings. First, the cross-sectional analysis of a single wave of KELS 2013 precludes conclusions about temporal ordering or causality. The indirect pathways therefore describe structural co-occurrence among variables, not mediation over time. This limitation is important because cross-sectional mediation analyses may misrepresent longitudinal mediation processes and cannot establish causal sequencing (Cole and Maxwell, 2003; Maxwell and Cole, 2007).

Second, the analytic sample was defined by gifted-program participation and a score of at least 22 out of 25 on the mathematics test. Findings should therefore not be generalized to all mathematically gifted learners, all gifted students, or all high-achieving mathematics students. The available sample may limit the precision of estimates from a model containing multiple latent constructs and indirect paths. This concern also applies to gender comparisons based on 137 boys and 101 girls. Non-significant estimates should not be interpreted as evidence that the corresponding associations are absent, particularly when confidence intervals allow substantively meaningful values. Characteristics and experiences of gifted learners may vary across identification procedures, educational programs, domains of achievement, and developmental stages (Neihart et al., 2021; Peterson et al., 2009; Pfeiffer, 2012). The 2014 data-collection period should also be considered when interpreting the results. Although mathematics achievement was used for sample selection, the psychological measures were not mathematics-specific. The findings therefore concern general beliefs and academic experiences within this selected subgroup and do not directly test psychological processes specific to mathematics or gifted education. Future studies should compare general and domain-specific measures within the same sample.

Third, sampling weights and school-level clustering were not incorporated in the AMOS models. The estimates should therefore be interpreted as model-based associations within the selected subgroup rather than design-based population estimates. Future studies should replicate the model using analytic approaches that account for the KELS sampling design.

Fourth, all major constructs were assessed through student self-reports, so shared method variance and response tendencies may have influenced the observed associations. Harman’s single-factor diagnostic cannot establish that common method bias is absent or small (Podsakoff et al., 2003). Future studies should combine student reports with parent reports, teacher ratings, or behavioral measures.

Fifth, the KELS measure captured cognitive and behavioral regulation but not motivational regulation. This is a limitation because self-regulated learning is commonly conceptualized as a multidimensional construct that includes cognitive, motivational, and behavioral regulation (Pintrich, 2000; Zimmerman, 2002, 2008). Because cognitive and behavioral regulation were represented through a common higher-order factor, the model did not estimate their separate associations with academic stress. Their distinct functions may have been obscured by this specification. In addition, excluding rehearsal narrowed the coverage of cognitive regulation. Future studies should examine these components separately, alongside broader measures of motivational regulation, to determine whether their associations with academic stress differ.

Finally, the AVE for parental support (0.448) was slightly below the 0.50 criterion, so convergent validity for this construct should be interpreted cautiously (Fornell and Larcker, 1981; Hair et al., 2010). Nevertheless, the standardized factor loadings and composite reliability values were acceptable, HTMT results supported discriminant validity, and the main findings were generally consistent across sensitivity analyses. Future work should distinguish emotional, academic, and autonomy support when examining students’ motivational, regulatory, and stress-related outcomes.

## 6 Conclusion

Among sixth-grade KELS 2013 students with gifted education experience who met the mathematics achievement criterion, perceived parental support was positively associated with growth mindset and self-regulated learning, and growth mindset was negatively associated with academic stress. A significant negative indirect association between parental support and academic stress was observed through growth mindset. Indirect associations involving self-regulated learning were not statistically significant. These findings describe general academic associations within the selected sample and identify relationships for further study. They do not establish temporal ordering, causal mechanisms, mathematics-specific processes, or the effectiveness of interventions.

## Data Availability

The data analyzed in this study is subject to the following licenses/restrictions: the data analyzed in this study are restricted-use secondary data from the Korean Education Longitudinal Study 2013, administered by the Korean Educational Development Institute. The authors are not permitted to redistribute the data. Eligible researchers may request access directly from KEDI through its research data request system: https://www.kedi.re.kr/khome/main/research/requestResData.do. Requests to access these datasets should be directed to Korean Educational Development Institute (KEDI) Research Data Request System: https://www.kedi.re.kr/khome/main/research/requestResData.do.

## References

[ref1] Bai B. Guo W. (2021). Motivation and self-regulated strategy use: relationships to primary school students' English writing in Hong Kong. Lang. Teach. Res. 25, 378–399. doi: 10.1177/1362168819859921

[ref2] Bai B. Wang J. Nie Y. (2021). Self-efficacy, task values and growth mindset: what has the most predictive power for primary school students’ self-regulated learning in English writing and writing competence in an Asian Confucian cultural context? Camb. J. Educ. 51, 65–84. doi: 10.1080/0305764X.2020.1778639

[ref3] Bermejo-Franco A. Sánchez-Sánchez J. L. Gaviña-Barroso M. I. Atienza-Carbonell B. Balanzá-Martínez V. Clemente-Suárez V. J. (2022). Gender differences in psychological stress factors of physical therapy degree students in the COVID-19 pandemic: a cross-sectional study. Int. J. Environ. Res. Public Health 19:810. doi: 10.3390/ijerph19020810, 35055632 PMC8776211

[ref4] Bezzina F. H. (2010). Investigating gender differences in mathematics performance and in self-regulated learning: an empirical study from Malta. Equal. Divers. Incl. Int. J. 29, 669–693. doi: 10.1108/02610151011074407

[ref5] Buff A. Reusser K. Dinkelmann I. (2017). Parental support and enjoyment of learning in mathematics: does change in parental support predict change in enjoyment of learning? ZDM 49, 423–434. doi: 10.1007/s11858-016-0823-7

[ref6] Chen F. F. (2007). Sensitivity of goodness of fit indexes to lack of measurement invariance. Struct. Equ. Model. Multidiscip. J. 14, 464–504. doi: 10.1080/10705510701301834

[ref7] Chen S. Jiang Y. Qiu S. Hu J. Wang L. Jiang Y. . (2025). Distinct roles of parental autonomy support and psychological control in Chinese elementary school students’ intelligence mindsets and academic outcomes. Asia-Pac. Educ. Res. 34, 133–146. doi: 10.1007/s40299-024-00842-5

[ref8] Cheung G. W. Rensvold R. B. (2002). Evaluating goodness-of-fit indexes for testing measurement invariance. Struct. Equ. Model. Multidiscip. J. 9, 233–255. doi: 10.1207/S15328007SEM0902_5

[ref9] Choe D. (2020). Parents’ and adolescents’ perceptions of parental support as predictors of adolescents’ academic achievement and self-regulated learning. Child Youth Serv. Rev. 116:105172. doi: 10.1016/j.childyouth.2020.105172

[ref10] Choi C. Lee J. Yoo M. S. Ko E. (2019). South Korean children's academic achievement and subjective well-being: the mediation of academic stress and the moderation of perceived fairness of parents and teachers. Child Youth Serv. Rev. 100, 22–30. doi: 10.1016/j.childyouth.2019.02.004

[ref11] Cohen J. (1992). Statistical power analysis. Curr. Dir. Psychol. Sci. 1, 98–101. doi: 10.1111/1467-8721.ep10768783

[ref12] Colarossi L. G. Eccles J. S. (2000). A prospective study of adolescents' peer support: gender differences and the influence of parental relationships. J. Youth Adolesc. 29, 661–678. doi: 10.1023/A:1026403922442

[ref13] Cole D. A. Maxwell S. E. (2003). Testing mediational models with longitudinal data: questions and tips in the use of structural equation modeling. J. Abnorm. Psychol. 112, 558–577. doi: 10.1037/0021-843X.112.4.558, 14674869

[ref14] Damrongpanit S. (2022). Effects of mindset, democratic parenting, teaching, and school environment on global citizenship of ninth-grade students. Eur. J. Educ. Res. 11, 217–230. doi: 10.12973/eu-jer.11.1.217

[ref15] Dan Q. Bai B. Huang Q. (2024). Gender differences in the relations between EFL students' classroom relationships and English language proficiency: the mediating role of self-regulated learning strategy use. Syst. 123:103311. doi: 10.1016/j.system.2024.103311

[ref16] Day A. L. Livingstone H. A. (2003). Gender differences in perceptions of stressors and utilization of social support among university students. Can. J. Behav. Sci. 35, 73–83. doi: 10.1037/h0087190

[ref17] de la Fuente J. Peralta-Sánchez F. J. Martínez-Vicente J. M. Sander P. Garzón-Umerenkova A. Zapata L. (2020). Effects of self-regulation vs. external regulation on the factors and symptoms of academic stress in undergraduate students. Front. Psychol. 11:1773. doi: 10.3389/fpsyg.2020.01773, 32982819 PMC7480135

[ref18] Dweck C. S. (1999). Self-Theories: Their Role in Motivation, Personality, and Development. New York: Psychology Press. doi: 10.4324/9781315783048

[ref19] Dweck C. S. (2006). Mindset: The New Psychology of Success. New York: Random House.

[ref20] Dweck C. S. Leggett E. L. (1988). A social-cognitive approach to motivation and personality. Psychol. Rev. 95, 256–273. doi: 10.1037/0033-295X.95.2.256

[ref21] Fornell C. Larcker D. F. (1981). Evaluating structural equation models with unobservable variables and measurement error. J. Mark. Res. 18, 39–50. doi: 10.1177/002224378101800104

[ref22] García-Moya I. Paniagua C. Jiménez-Iglesias A. (2025). Gender differences in adolescent school stress: a mixed-method study. J. Res. Adolesc. 35:e13057. doi: 10.1111/jora.13057, 40040599

[ref23] García-Ros R. Pérez-González F. Tomás J. M. Sancho P. (2023). Effects of self-regulated learning and procrastination on academic stress, subjective well-being, and academic achievement in secondary education. Curr. Psychol. 42, 26602–26616. doi: 10.1007/s12144-022-03759-8

[ref24] Graves B. S. Hall M. E. Dias-Karch C. Haischer M. H. Apter C. (2021). Gender differences in perceived stress and coping among college students. PLoS One 16:e0255634. doi: 10.1371/journal.pone.0255634, 34383790 PMC8360537

[ref25] Hair J. F. Jr. Black W. C. Babin B. J. Anderson R. E. (2010). Multivariate Data Analysis: A Global Perspective. 7th Edn Boston: Pearson.

[ref26] Han S. W. Borgonovi F. (2026). Growth mindsets and achievement: evidence across academic and creative domains from an international perspective. J. Adolesc. 98, 1064–1074. doi: 10.1002/jad.70117, 41636320

[ref27] Harman H. H. (1976). Modern Factor Analysis. Chicago: University of Chicago Press.

[ref28] Henseler J. Ringle C. M. Sarstedt M. (2015). A new criterion for assessing discriminant validity in variance-based structural equation modeling. J. Acad. Mark. Sci. 43, 115–135. doi: 10.1007/s11747-014-0403-8

[ref29] Hill A. P. Madigan D. J. (2022). Perfectionism, mattering, stress, and self-regulation of home learning of UK gifted and talented students during the COVID-19 pandemic. Gift. Talent. Int. 37, 56–63. doi: 10.1080/15332276.2022.2033649

[ref30] Högberg B. Strandh M. Hagquist C. (2020). Gender and secular trends in adolescent mental health over 24 years–the role of school-related stress. Soc. Sci. Med. 250:112890. doi: 10.1016/j.socscimed.2020.112890, 32143086

[ref31] Hu L. T. Bentler P. M. (1999). Cutoff criteria for fit indexes in covariance structure analysis: conventional criteria versus new alternatives. Struct. Equ. Model. Multidiscip. J. 6, 1–55. doi: 10.1080/10705519909540118

[ref32] Jung J. Y. Lee J. (2024). Parental attitudes toward gifted students and gifted education: attitude profiles and predictors. J. Intelligence 12:48. doi: 10.3390/jintelligence12050048, 38786650 PMC11122637

[ref34] Kim J. J. Fung J. Wu Q. Fang C. Lau A. S. (2017). Parenting variables associated with growth mindset: an examination of three Chinese-heritage samples. Asian Am. J. Psychol. 8, 115–125. doi: 10.1037/aap0000064

[ref33] Kim K. M. (2021). What makes adolescents psychologically distressed? Life events as risk factors for depression and suicide. Eur. Child Adolesc. Psychiatry 30, 359–367. doi: 10.1007/s00787-020-01520-9, 32232580

[ref35] Kristjánsson Á. L. Sigfúsdóttir I. D. (2009). The role of parental support, parental monitoring, and time spent with parents in adolescent academic achievement in Iceland: a structural model of gender differences. Scand. J. Educ. Res. 53, 481–496. doi: 10.1080/00313830903180786

[ref36] Kulakow S. Raufelder D. Hoferichter F. (2021). School-related pressure and parental support as predictors of change in student stress levels from early to middle adolescence. J. Adolesc. 87, 38–51. doi: 10.1016/j.adolescence.2020.12.008, 33453550

[ref37] Lazarus R. S. Folkman S. (1984). Stress, Appraisal, and Coping. New York: Springer Publishing Company.

[ref39] Lee J. Yu H. Choi S. (2012). The influences of parental acceptance and parental control on school adjustment and academic achievement for south Korean children: the mediation role of self-regulation. Asia Pac. Educ. Rev. 13, 227–237. doi: 10.1007/s12564-011-9186-5

[ref38] Lee M. Larson R. (2000). The Korean ‘examination hell’: long hours of studying, distress, and depression. J. Youth Adolesc. 29, 249–271. doi: 10.1023/A:1005160717081

[ref40] Malespina A. Schunn C. D. Singh C. (2022). Whose ability and growth matter? Gender, mindset and performance in physics. Int. J. STEM Educ. 9:28. doi: 10.1186/s40594-022-00342-2

[ref41] Marsch S. Yanagida T. Steinberg E. (2024). Workplace learning: the bidirectional relationship between stress and self-regulated learning in undergraduates. BMC Med. Educ. 24:1038. doi: 10.1186/s12909-024-06021-w, 39334175 PMC11429500

[ref42] Maxwell S. E. Cole D. A. (2007). Bias in cross-sectional analyses of longitudinal mediation. Psychol. Methods 12, 23–44. doi: 10.1037/1082-989X.12.1.23, 17402810

[ref43] Mosanya M. (2021). Buffering academic stress during the COVID-19 pandemic related social isolation: grit and growth mindset as protective factors against the impact of loneliness. Int. J. Appl. Posit. Psychol. 6, 159–174. doi: 10.1007/s41042-020-00043-7, 33083519 PMC7561702

[ref44] Neihart M. Pfeiffer S. I. Cross T. L. (2021). The Social and Emotional Development of Gifted Children: What Do We Know? 2nd Edn New York: Routledge.

[ref45] Nolen-Hoeksema S. (2012). Emotion regulation and psychopathology: the role of gender. Annu. Rev. Clin. Psychol. 8, 161–187. doi: 10.1146/annurev-clinpsy-032511-143109, 22035243

[ref46] Park B. H. Choi M. G. Kim N. R. (2011). The difference of home environment and self-regulated learning between gifted students and general students in elementary school [in Korean]. J. Korean Soc. Gifted Talented 10, 189–211. doi: 10.17839/jksgt.2011.10.2.189

[ref47] Peterson J. S. (2009). Myth 17: gifted and talented individuals do not have unique social and emotional needs. Gift. Child Q. 53, 280–282. doi: 10.1177/0016986209346946

[ref48] Peterson J. Duncan N. Canady K. (2009). A longitudinal study of negative life events, stress, and school experiences of gifted youth. Gift. Child Q. 53, 34–49. doi: 10.1177/0016986208326553

[ref49] Pfeiffer S. I. (2012). Serving the Gifted: Evidence-Based, Clinical and Psychoeducational Practice. New York: Routledge.

[ref50] Pintrich P. R. (2000). “The role of goal orientation in self-regulated learning,” in Handbook of Self-Regulation, eds. Boekaerts M. Pintrich P. R. Zeidner M. (San Diego, CA: Academic Press), 451–502.

[ref51] Pintrich P. R. De Groot E. V. (1990). Motivational and self-regulated learning components of classroom academic performance. J. Educ. Psychol. 82, 33–40. doi: 10.1037/0022-0663.82.1.33

[ref52] Podsakoff P. M. MacKenzie S. B. Lee J. Y. Podsakoff N. P. (2003). Common method biases in behavioral research: a critical review of the literature and recommended remedies. J. Appl. Psychol. 88, 879–903. doi: 10.1037/0021-9010.88.5.879, 14516251

[ref53] Ringeisen T. Raufelder D. (2015). The interplay of parental support, parental pressure and test anxiety–gender differences in adolescents. J. Adolesc. 45, 67–79. doi: 10.1016/j.adolescence.2015.08.018, 26378971

[ref54] Rose A. J. Rudolph K. D. (2006). A review of sex differences in peer relationship processes: potential trade-offs for the emotional and behavioral development of girls and boys. Psychol. Bull. 132, 98–131. doi: 10.1037/0033-2909.132.1.98, 16435959 PMC3160171

[ref55] Shin M. Ahn D. (2014). Factors influencing self-regulated strategies: on autonomy support and beliefs of intelligence ability of gifted and non-gifted students [in Korean]. J. Gift. Talented Educ. 24, 877–892. doi: 10.9722/JGTE.2014.24.5.877

[ref56] Toraman Ç. Aktan O. Korkmaz G. (2022). How can we make students happier at school? Parental pressure or support for academic success, educational stress, and school happiness of secondary school students. Shanlax Int. J. Educ. 10, 92–100. doi: 10.34293/education.v10i2.4546

[ref57] Tsai M. Y. (2023). Comparing perfectionism, cognitive mindset, constructive thinking, and emotional intelligence in gifted students by grade and gender. Soc. Sci. 12:233. doi: 10.3390/socsci12040233

[ref58] Unger D. G. McLeod L. E. Brown M. B. Tressell P. A. (2000). The role of family support in interparental conflict and adolescent academic achievement. J. Child Fam. Stud. 9, 191–202. doi: 10.1023/A:1009419005136 11033927

[ref59] Wang Y. Tang X. Wu S. Sun X. (2026). Growth mindsets are less endorsed and less associated with academic performance in non-WEIRD cultures. npj Sci. Learn. 11:21. doi: 10.1038/s41539-026-00406-w, 41730903 PMC13039518

[ref60] West S. G. Finch J. F. Curran P. J. (1995). “Structural equation models with nonnormal variables: problems and remedies,” in Structural Equation Modeling: Concepts, Issues, and Applications, ed. Hoyle R. H. (Thousand Oaks, CA: Sage), 56–75.

[ref61] Wolf E. J. Harrington K. M. Clark S. L. Miller M. W. (2013). Sample size requirements for structural equation models: an evaluation of power, bias, and solution propriety. Educ. Psychol. Meas. 73, 913–934. doi: 10.1177/0013164413495237, 25705052 PMC4334479

[ref62] Yao Y. Zhu X. Zhan J. (2024). The associations among growth mindsets, the ideal L2 writing self, and L2 writing enjoyment and their impacts on L2 English writing performance: a gender difference perspective. Assess. Writ. 60:100832. doi: 10.1016/j.asw.2024.100832

[ref63] Zhao H. Xiong J. Zhang Z. Qi C. (2021). Growth mindset and college students’ learning engagement during the COVID-19 pandemic: a serial mediation model. Front. Psychol. 12:621094. doi: 10.3389/fpsyg.2021.621094, 33679536 PMC7933026

[ref64] Zhao S. Zhang Y. Yu C. Zhang H. Xie M. Chen P. . (2023). Trajectories of perceived stress among students in transition to college: mindset antecedents and adjustment outcomes. J. Youth Adolesc. 52, 1873–1886. doi: 10.1007/s10964-023-01788-5, 37296270 PMC10255944

[ref65] Zimmerman B. J. (2002). Becoming a self-regulated learner: an overview. Theory Pract. 41, 64–70. doi: 10.1207/s15430421tip4102_2

[ref66] Zimmerman B. J. (2008). Investigating self-regulation and motivation: historical background, methodological developments, and future prospects. Am. Educ. Res. J. 45, 166–183. doi: 10.3102/0002831207312909

