# Supplementary material for: Growth mindset as a structural link between parental support and academic stress among mathematically high-achieving elementary students with gifted education experience
Source: Front Psychol. 2026 Oct 16;17:1954883. doi: 10.3389/fpsyg.2026.1954883 (PMC13719541; doi:10.3389/fpsyg.2026.1954883)
Supplement: Supplementary file 1 [file Data_sheet_1.pdf]

Supplementary Materials

A confirmatory factor analysis was conducted to examine the parental support scale. Among the original eight items, Items 5, 6, and 7 showed standardized factor loadings below .50 and were removed. Item 1 was additionally excluded because its content reflected a broader perception of the home educational environment rather than specific parental support behaviors. The final four-item scale included Items 2, 3, 4, and 8. The results are presented in Supplementary Table S1.

Supplementary Table S1. Model fit, validity, and reliability indices for alternative specifications of the perceived parental support scale.

Scale specification	χ^2	*df*	*p*	CFI	TLI	RMSEA	SRMR	AVE	CR	Cronbach's α
Original eight-item scale	112.437	20	< .001	.798	.717	.140	.090	.320	.785	.784
Five-item scale after removing Items 5, 6, and 7	15.866	5	.007	.963	.925	.096	.040	.418	.779	.776
Final four-item scale after removing Items 1, 5, 6, and 7	3.186	2	.203	.995	.984	.050	.020	.449	.762	.757

Note. *N* = 238, CFI = comparative fit index; TLI = Tucker–Lewis index; RMSEA = root mean square error of approximation; SRMR = standardized root mean square residual; AVE = average variance extracted; CR = composite reliability. Recommended cutoffs were CFI and TLI ≥ .90, RMSEA and SRMR ≤ .08, AVE ≥ .50, and CR and Cronbach's α ≥ .70. p values below .001 are reported as < .001. The estimates in this table were obtained from scale-specific CFAs and may differ slightly from the corresponding estimates obtained from the overall measurement model reported in Table 4 of the main manuscript.

Because the three-item single-factor CFA for the growth mindset scale was saturated (df = 0), global fit indices were not reported. AVE, composite reliability, and Cronbach's α are reported in Supplementary Table S2.

Supplementary Table S2. Measurement indices for the three-item growth mindset scale.

Scale	Number of items	AVE	CR	Cronbach's α
Growth mindset	3	.676	.861	.855

Note. N = 238. The three-item single-factor model was saturated (df = 0); therefore, global fit indices were not reported. AVE = average variance extracted; CR = composite reliability. The estimates in this table were obtained from the scale-specific CFA and therefore differ slightly from the corresponding estimates obtained from the overall measurement model reported in Table 4 of the main manuscript.

Confirmatory factor analyses were conducted for the cognitive regulation and behavioral regulation subscales. The cognitive regulation model showed acceptable fit. In the behavioral regulation model, one item from the help-seeking and resource use factor showed a standardized factor loading below .50 and was removed. The revised 19-item behavioral regulation model showed acceptable fit. The results are presented in Supplementary Table S3.

Supplementary Table S3. Confirmatory factor analysis fit indices for the cognitive and behavioral regulation strategy scales before and after item deletion.

Scale specification	χ^2	*df*	*p*	CFI	TLI	RMSEA	SRMR
Cognitive regulation strategy scale: 12 items	95.922	48	< .001	.967	.955	.065	.057
Behavioral regulation strategy scale before item deletion: 20 items	361.900	160	< .001	.920	.904	.073	.051
Behavioral regulation strategy scale after item deletion: 19 items	286.789	142	< .001	.940	.928	.066	.046

Note. $N = 238$. In the behavioral regulation strategy scale, one item from the help-seeking and resource-use subscale was removed because its standardized factor loading was below .50. CFI = comparative fit index; TLI = Tucker–Lewis index; RMSEA = root mean square error of approximation; SRMR = standardized root mean square residual. Recommended cutoffs were CFI and TLI ≥ .90 and RMSEA and SRMR ≤ .08. p values below .001 are reported as < .001.

Supplementary Table S4. AVE, composite reliability, and internal consistency estimates for cognitive regulation subscales.

Subscale	Number of items	AVE	CR	Cronbach's α
Rehearsal	3	.593	.811	.800
Elaboration	3	.624	.830	.817
Organization	3	.537	.777	.778
Metacognition	3	.695	.872	.866

Note. N = 238. These estimates were calculated at the subscale level. Although the rehearsal subscale showed acceptable reliability at the subscale level, it was excluded from the final self-regulated learning construct because its standardized factor loading in the overall measurement model was below .50. AVE = average variance extracted; CR = composite reliability. Recommended cutoffs were AVE ≥ .50 and CR and Cronbach's α ≥ .70.

Supplementary Table S5. AVE, composite reliability, and internal consistency estimates for behavioral regulation subscales before item deletion: 20-item scale.

Subscale	Number of items	AVE	CR	Cronbach's α
Academic performance regulation	5	.618	.890	.886
Effort regulation	3	.644	.844	.841
Time management	3	.596	.816	.814
Space management	2	.588	.728	.674
Help-seeking and resource use	7	.449	.848	.837

Note. *N* = 238. Before item deletion, the AVE for the help-seeking and resource use subscale was .449, which was below the recommended cutoff of .50. Cronbach's α for the two-item space management subscale was .674, slightly below the recommended cutoff of .70, although its AVE and CR were acceptable. AVE = average variance extracted; CR = composite reliability.

frontiers

Supplementary Table S6. AVE, composite reliability, and internal consistency estimates for behavioral regulation subscales after item deletion: 19-item scale.

Subscale	Number of items	AVE	CR	Cronbach's α
Academic performance regulation	5	.618	.890	.886
Effort regulation	3	.644	.844	.841
Time management	3	.596	.816	.814
Space management	2	.590	.729	.674
Help-seeking and resource use	6	.496	.855	.846

Note. *N* = 238. After item deletion, the AVE for the help-seeking and resource-use subscale was .496, remaining slightly below the recommended cutoff of .50; therefore, this subscale should be interpreted with caution. Cronbach's α for the two-item space management subscale was .674, slightly below the recommended cutoff of .70, although its AVE and CR were acceptable. AVE = average variance extracted; CR = composite reliability.

frontiers

The initial four-item, one-factor model of academic stress yielded $\chi^2(2) = 7.151$, $p = .028$, CFI = .984, TLI = .952, and RMSEA = .104. Following inspection of the initial model fit, a residual covariance between the third and fourth retained items (original Items 3 and 4) was added as a post hoc modification. Both items describe negative responses to thoughts about studying: digestive discomfort in Item 3 and reduced enthusiasm for life when contemplating further years of study in Item 4. This shared emphasis provides a possible content-based explanation for residual covariance beyond the common academic stress factor. The revised model yielded $\chi^2(1) = 0.619$, $p = .431$, CFI = 1.000, TLI = 1.007, and RMSEA = .000. Results for the revised scale-specific CFA are presented in Supplementary Table S7. This residual covariance was included only in the scale-specific CFA; it was not specified in the overall measurement or structural models.

Supplementary Table S7. Confirmatory factor analysis fit, validity, and reliability indices for the academic stress scale.

Scale	χ^2	*df*	*p*	CFI	TLI	RMSEA	SRMR	AVE	CR	Cronbach's α
Academic stress	.619	1	.431	1.000	1.007	.000	.007	.521	.812	.819

Note. *N* = 238. CFI = comparative fit index; TLI = Tucker–Lewis index; RMSEA = root mean square error of approximation; SRMR = standardized root mean square residual; AVE = average variance extracted; CR = composite reliability. Recommended cutoffs were CFI and TLI ≥ .90, RMSEA and SRMR ≤ .08, AVE ≥ .50, and CR and Cronbach's α ≥ .70. The estimates in this table were obtained from the scale-specific CFA and therefore differ slightly from those obtained from the overall measurement model reported in Table 4 of the main manuscript.

The overall measurement model including parental support, growth mindset, self-regulated learning, and academic stress was examined. The rehearsal factor showed a standardized factor loading below .50 in the overall measurement model and was therefore excluded from the final measurement model. Supplementary Table S8 presents model fit before and after excluding the rehearsal factor.

Supplementary Table S8. Overall measurement-model fit before and after excluding the rehearsal factor from the self-regulated learning construct

Model	χ^2	*df*	*p*	CFI	TLI	RMSEA	SRMR
Before excluding the rehearsal factor	287.818	163	< .001	.943	.933	.057	.058
After excluding the rehearsal factor	262.872	145	< .001	.945	.935	.059	.055

Note. *N* = 238. CFI = comparative fit index; TLI = Tucker–Lewis index; RMSEA = root mean square error of approximation; SRMR = standardized root mean square residual. Recommended cutoffs were CFI and TLI ≥ .90 and RMSEA and SRMR ≤ .08. *p* values below .001 are reported as < .001. The residual variance of behavioral regulation was fixed at .00001 in the final measurement model.

frontiers

Supplementary Table S9. Sensitivity analyses of model fit, key paths, and indirect associations across alternative model specifications.

Model	Alternative specification	*N*	CFI	TLI	RMSEA	SRMR	PS→GM (β)	GM→AS (β)	PS→GM→AS (B)	95% CI
Main model	Four-item parental support scale; rehearsal excluded; math score ≥ 22	238	.941	.930	.058	.061	.246**	-.296***	-.090**	[-.234, -.021]
Sensitivity 1	Five-item parental support scale	238	.931	.920	.060	.064	.252**	-.295***	-.087**	[-.232, -.019]
Sensitivity 2	Original eight-item parental support scale	238	.882	.865	.072	.079	.222**	-.267**	-.072*	[-.212, -.008]
Sensitivity 3	Rehearsal factor included in SRL	238	.936	.926	.057	.063	.246**	-.298***	-.090**	[-.235, -.021]
Sensitivity 4	More restrictive math score criterion ≥ 23	186	.939	.928	.060	.066	.203*	-.276**	-.064*	[-.211, -.003]

Note. $N = 238$ for the main model and Sensitivity Models 1–3; $N = 186$ for Sensitivity Model 4. $^{*}p < .05$, $^{**}p < .01$, $^{***}p < .001$. PS = parental support; GM = growth mindset; SRL = self-regulated learning; AS = academic stress; CI = confidence interval. The main model corresponds to the hypothesized model reported in Tables 6 and 7 in the main text. Sensitivity models examined alternative parental support scales, inclusion of the rehearsal factor in SRL, and a more restrictive mathematics achievement criterion. Direct path coefficients are standardized coefficients (β), whereas indirect associations are unstandardized estimates (B) with 95% bias-corrected bootstrap confidence intervals based on 5,000 resamples.
